# Clinical Factors for Severity of *Plasmodium falciparum* Malaria in Hospitalized Adults in Thailand

**DOI:** 10.1371/journal.pone.0071503

**Published:** 2013-08-12

**Authors:** Patrick Sagaki, Vipa Thanachartwet, Varunee Desakorn, Duangjai Sahassananda, Supat Chamnanchanunt, Wirongrong Chierakul, Punnee Pitisuttithum, Prajej Ruangkanchanasetr

**Affiliations:** 1 Department of Clinical Tropical Medicine, Faculty of Tropical Medicine, Mahidol University, Bangkok, Thailand; 2 Information Technology Unit, Faculty of Tropical Medicine, Mahidol University, Bangkok, Thailand; 3 Amudat Hospital, Amudat, Uganda; 4 Department of Medicine, Phramongkutklao Hospital and college of Medicine, Bangkok, Thailand; Universidade Federal de Minas Gerais, Brazil

## Abstract

*Plasmodium falciparum* is a major cause of severe malaria in Southeast Asia, however, there is limited information regarding clinical factors associated with the severity of falciparum malaria from this region. We performed a retrospective case-control study to compare clinical factors and outcomes between patients with severe and non-severe malaria, and to identify clinical factors associated with the requirement for intensive care unit (ICU) admission of patients with severe falciparum malaria among hospitalized adults in Southeast Asia. A total of 255 patients with falciparum malaria in the Hospital for Tropical Diseases in Bangkok, Thailand between 2006 and 2012 were included. We identified 104 patients with severe malaria (cases) and 151 patients with non-severe malaria (controls). Patients with falciparum malaria with following clinical and laboratory characteristics on admission (1) referrals, (2) no prior history of malaria, (3) body temperature of >38.5°C, (4) white blood cell counts >10×10^9^/µL, (5) presence of schizonts in peripheral blood smears, and (6) albumin concentrations of <3.5 g/dL, were more likely to develop severe malaria (*P*<0.05). Among patients with severe malaria, patients who met ≥3 of the 2010 WHO criteria had sensitivity of 79.2% and specificity of 81.8% for requiring ICU admission. Multivariate analysis identified the following as independent associated factors for severe malaria requiring ICU admission; (1) ethnicity of Thai [odds ratio (OR) = 3.601, 95% confidence interval (CI) = 1.011–12.822] or Myanmar [OR = 3.610, 95% CI = 1.138–11.445]; (2) referrals [OR = 3.571, 95% CI = 1.306–9.762]; (3) no prior history of malaria [OR = 5.887, 95% CI = 1.354–25.594]; and (4) albumin concentrations of <3.5 g/dL [OR = 7.200, 95% CI = 1.802–28.759]. Our findings are important for the clinical management of patients with malaria because it can help early identification of patients that could develop severe malaria and require ICU admission. Early identification and the timely initiation of appropriate treatments may well improve the outcomes and reduce the mortality of these patients.

## Introduction

Malaria is caused by various species of the protozoan parasite *Plasmodium,* namely *P. falciparum*, *P. vivax*, *P. malariae, P. ovale* and *P*. *knowlesi*. Of these, *P. falciparum* and *P. vivax* are responsible for the majority of infections [1]. Malaria is endemic in 106 countries, and the global death rate due to malaria infection is estimated at 1 million individuals per annum [1]. Approximately 40% of the global population at risk of contracting malaria resides in Southeast Asia. In Thailand, 32 million people are at risk of contracting malaria, and the Thai-Cambodia border is known to be an area with a high transmission rate, especially for multi-drug resistant *P. falciparum* malaria [2]. *P. falciparum* is a major cause of severe malaria and approximately 10–20% of patients with falciparum malaria require urgent and intensive medical care [3]–[5].

The World Health Organization (WHO) provides case definitions for malaria infection as well as guidance on the management of malaria and for severe malaria *per se* [1], [6]. A previous study reported that mortality rates for severe malaria (as defined by WHO) increase from 9.5% in patients who meet 1 of the criteria that defines severe malaria to 50% in patients who meet ≥5 of these criteria [7]. Thus far, only a few studies have investigated clinical factors that predict severity in patients with falciparum malaria [8]–[10]. In addition, information on WHO criteria that associated with the severity in patients with falciparum malaria in Southeast Asia is limited [4], [5], [7]. Therefore, we conducted a retrospective case-control study to compare clinical factors and outcomes between patients with severe falciparum malaria and those with non-severe falciparum malaria, and to identify clinical factors associated with the requirement for intensive care unit (ICU) admission in patients with severe falciparum malaria among hospitalized adults in Thailand.

## Materials and Methods

The Standards for the Reporting of Observational Studies in Epidemiology (STROBE) procedure were followed in this study.

### Study Design and Population

The study protocol was approved by the Ethics Committee of the Faculty of Tropical Medicine, Mahidol University in Bangkok, Thailand. The written consent was not obtained and it was specifically waived by the approving ethic committee of the Faculty of Tropical Medicine. This was a retrospective case-control study conducted among adults admitted at the Hospital for Tropical Diseases in Bangkok, Thailand between 2006 and 2012. Patients who met the criteria for having severe falciparum malaria defined by the 2010 WHO criteria (cases) and patients who did not meet these criteria (controls) were identified from a prospective cohort of 300 patients with falciparum malaria. Patients aged ≥15 years and diagnosed with falciparum malaria by presence of asexual *P. falciparum* forms in thin or thick peripheral blood smear were included in our study. Patients who had mixed infections with other forms of *Plasmodium* species, community acquired infection, dengue, rickettsiosis and leptospirosis; pregnancy; and underlying medical illness were excluded. In the Hospital for Tropical Diseases, blood culture for microbiology, urinalysis, and chest X-ray were routinely performed in hospitalized patients with malaria in order to exclude other infections. In addition, serological testing for diagnosis of dengue, rickettsiosis and leptospirosis were performed when clinical findings were suspected.

**Figure 1:**
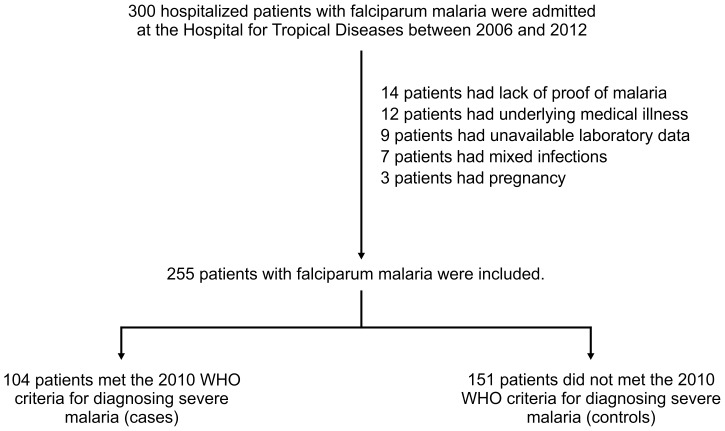
Patient recruitment flow chart.

The demographic information, clinical and laboratory findings, and patient outcomes were transferred into a pre-defined case record-form. Medical records and discharge summaries of the 300 patients with a diagnosis of falciparum malaria at the time of death or discharge from hospital were summarized and classified as cases and controls. Of the 300 patients with falciparum malaria, 45 patients were excluded because 14 patients had a lack of proof of malarial infection, 9 patients did not have available laboratory data, 7 patients had evidence of mixed infections, 12 patients had underlying medical diseases, and 3 were pregnant (Figure 1).

The remaining 255 patients with falciparum malaria were included. Using the 2010 WHO criteria for diagnosing severe malaria, 104 patients met the criteria for severe malaria defined as cases and the remainder (n  = 151) were classified as controls. Among 104 patients with severe falciparum malaria, 49 patients were admitted at the ICU and 55 patients were admitted at the wards.

### Case Definition of Severe Malaria According to the 2010 WHO Criteria

In our study, patients with falciparum malaria were considered to have had severe malaria if they presented with ≥1 of the following clinical or laboratory findings: (1) impaired consciousness, defined by a Glasgow Coma Score (GCS) of <10; (2) multiple convulsions, defined as >2 episodes of convulsions in 24 hours; (3) acidotic breathing; (4) shock, defined by a systolic blood pressure of <80 mmHg or the need for inotropic drugs; (5) clinical jaundice with evidence of other vital organ dysfunction including acute kidney injury defined by increase of serum creatinine ≥0.3 mg/dl within 48 hours, shock, alteration of consciousness defined by GCS <15, and hypoxemia defined by oxygen saturation <93% in room air; (6) dark colored urine; (7) spontaneous bleeding, and 8) pulmonary edema detected by chest X-ray. The laboratory findings that indicated severe malaria included: (1) hypoglycemia, defined as blood sugar levels of <40 mg/dL; (2) severe metabolic acidosis, defined as serum bicarbonate levels of <15 mmol/L; (3) severe anemia, defined as hemoglobin (Hb) levels of <5 g/dL; (4) hemoglobinuria, defined as positive hemoglobin with the presence of ≤2 red blood cells per high power field in urine; (5) hyperparasitemia, defined as >2%/100,000/µL parasites in a blood smear in areas with low intensity transmission, or >5%/250,000/µL parasites in a blood smear in high, stable malaria transmission areas; and (6) renal impairment, defined as serum creatinine levels of >3 mg/dL. Time to febrile clearance was defined as the time (in hours) from the initial treatment dose of an anti-malarial drug to the start of the first sustained decrease in oral temperature of ≤37.5°C for a period of ≥48 h. Time to parasite clearance was defined as the time (in hours) from the initial treatment dose of an anti-malarial drug to the first of two consecutive peripheral blood smears with no evidence of parasitemia. Community acquired infection is defined as any infection diagnosed within 48 hours of hospitalization and hospital acquired infection is defined as any infection diagnosed after 48 hours of hospitalization.

### Sample Size Calculation

The study sample size was estimated with the use of the Power and Sample Size Program, version 3.0, 2009 (Dupont WD, Creative Commons Attribution-Non Commercial-NoDerivs 3.0 United States License). We planned a study with a ratio of 1∶1.5 for independent cases of severe malaria to control patients. The probability that control patients could have had severe malaria was estimated at 0.2. If the true odds ratio was 2.25 for severe malaria relative to non-severe malaria, then we had to include 101 cases of severe malaria and 152 control patients to be able to reject a null hypothesis if the odds ratio equaled 1. The power of the study was set at 80% and the Type I error probability associated with a null hypothesis were 0.05.

### Statistical Analysis

All data analyses were performed with SPSS for Windows 18.0 (SPSS Inc. Chicago, IL). Categorical variables were compared with Chi-square analyses or Fisher’s exact test where appropriate, and data for these variables are summarized as number and percentage. We used the Kolgomorov Smirnov test to ascertain whether numerical variables were normally distributed. All variables with normal distributions were compared with Student *t*-tests and the data for these variables are reported as mean ± standard deviation (SD). Variables that did not pass the normality test were compared with Mann–Whitney U tests for comparison of 2 groups and data for these variables are reported as median and inter-quartile range (IQR). Univariate analysis was performed to determine possible factors that could be associated with severe malaria. All variables with *P*≤0.2 in the univariate logistic regression analysis was included in the stepwise multiple logistic regression analysis by using the backward selection method for determining independent associated factors for severe malaria. All tests of significance were 2-sided, and *P*<0.05 was considered statistically significant. Diagnostic sensitivity and specificity of the 2010 WHO criteria was defined against those requiring ICU admission, and was expressed as a proportion with a 95% confidence interval (CI).

## Results

Of the 255 patients diagnosed with falciparum malaria, 104 met the 2010 WHO criteria for severe malaria defined as cases and the remainder of the patients (n  = 151) were included in the control group.

### Comparison between Patients with Severe and Non-severe Falciparum Malaria

There were no significant differences in the age, gender and ethnicity among the patients with and without severe malaria. There was also no significant difference in the number of patients with and without severe malaria who visited to the Thai-Myanmar border or other areas. Patients with severe malaria were significantly more likely to have been referred for treatment to the Hospital for Tropical Diseases in Bangkok and had no a prior history of malaria. All statistical comparisons for baseline characteristics of patients with or without severe malaria are reported in table 1.

**Table 1:** Baseline characteristics, clinical parameters, management and outcomes of hospitalized patients with falciparum malaria (severe or non-severe malaria).

	Falciparum malaria					
Characteristics	Severe (cases)		Non-severe (controls)		*P*-value	
	n	no. (%)	n	no. (%)		
*Baseline characteristics*						
Age, median (IQR), years	104	26.0 (19.0–36.0)	151	26.0 (21.0–35.0)	0.617	
Gender (male)	104	73 (70.2)	151	123 (81.5)	0.052	
Ethnic group	104		151		0.121	
Thai		29 (27.9)		61 (40.4)		
Myanmar		37 (35.6)		44 (29.1)		
Others		38 (36.6)		46 (30.5)		
Area visited	92		126		0.868	
Thai-Myanmar border		85 (92.4)		116 (92.1)		
Others		7 (7.6)		10 (7.9)		
Referrals	104	51 (49.0)	149	25 (16.8)	<0.001	
No history of malaria	103	81 (78.6)	150	84 (56.0)	0.001	
*Clinical and laboratory findings*						
Temperature >38.5°C	104	65 (62.5)	151	68 (45.0)	0.009	
WBC >10×10^9^/µL	104	29 (27.9)	151	12 (7.9)	<0.001	
Presence of schizonts	104	47 (45.2)	151	14 (9.3)	<0.001	
Albumin <3.5 g/dL	104	77 (74.0)	151	47 (31.1)	<0.001	
*Management and outcomes*						
Total artesunate, median (IQR), mg	104	840 (652–980)	151	600 (600–600)	<0.001	
Total artesunate, median (IQR), mg/kg	79	14.4 (11.2–18.2)	141	11.1 (9.3–13.1)	<0.001	
FCT, median (IQR), hours	103	93.0 (60.0–129.0)	150	50 (33.2–81.4)	<0.001	
PCT, median (IQR), hours	103	77.0 (58.7–101.7)	149	56.0 (43.8–74.6)	<0.001	
Hospitalization, median (IQR), days	104	7.5 (6.0–11.0)	151	5.0 (4.0–6.0)	<0.001	
Hospital acquired infection	104	45 (43.3)	151	3 (2.0)	<0.001	

**Note:** IQR = interquartile range; WBC = white blood cell counts; FCT = fever clearance time; PCT = parasite clearance time.

All of the patients with falciparum malaria presented with fever at the time of admission. Patients with severe malaria were significantly more likely to have had temperature >38.5°C, white blood cell counts >10×10^9^/µL, presence of schizonts in the peripheral blood smears, and albumin concentration of <3.5 g/dL at the time of admission (Table 1). All patients were treated with an artesunate-based regimen. Patients with severe malaria received higher total dosages of artesunate and higher adjusted total dosages of artesunate per kilogram of body weight. Patients with severe malaria also had significantly longer fever and parasite clearance times, and periods of hospitalization than patients with non-severe malaria. In addition, patients with severe malaria had higher proportion of hospital acquired infections, but there was no in-hospital mortality in this study (Table 1).

### Characteristics of Patients with Severe Falciparum Malaria

Of the 255 patients diagnosed with falciparum malaria, 104 met the 2010 WHO criteria for severe malaria. In 67 (64.4%) patients, a clinical diagnosis of severe malaria was made because of the presence of clinical jaundice with evidence of other vital organ dysfunction including acute kidney injury (53 patients, 79.1%), shock (21 patients, 31.3%), alteration of consciousness (17 patients, 25.4%), and hypoxemia (15 patients, 22.4%); in 27 (26.0%) because of evidence of shock; in 21 (20.2%) because of pulmonary edema; in 18 (17.3%) because of dark colored urine excretion; in 14 (13.5%) because of impaired consciousness; in 6 (5.8%) because of abnormal spontaneous bleeding; and in 6 (5.8%) because of an acidotic breath. Laboratory findings that indicated a diagnosis of severe malaria included abnormally high hemoglobinuria in 51 (49.0%) patients; hyperparasitemia in 44 (42.3%); renal impairment in 20 (19.2%); severe metabolic acidosis in 17 (16.3%); severe anemia in 3 (2.9%); and hypoglycemia in 2 (1.9%). Majority of clinical and laboratory diagnosis of severe malaria was found at admission, but some of the patients with renal failure (5/20 patients, 25%), shock (4/27 patients, 14.8%), pulmonary edema (2/21 patients, 9.5%) were diagnosed after hospitalization.

The sensitivity of 2010 WHO criteria for diagnosing severe malaria, for requiring ICU admission was low, but the specificity of the criteria for requiring ICU admission was high with the exception that the criteria of jaundice with evidence of other vital organ dysfunction, hemoglobinuria, and hyperparasitemia had lower specificity. However, severe falciparum malaria patients who met ≥3 of the 2010 WHO criteria had sensitivity (95% CI) of 79.2% (64.6–89.0) and specificity (95% CI) of 81.8% (68.7–90.5) for requiring ICU admission (Table 2).

**Table 2:** Sensitivity and specificity of the 2010 WHO criteria for requiring intensive care unit in hospitalized patients with severe falciparum malaria.

2010 WHO Criteria		ICU	Without ICU	% (95% Confidence Interval)	
		admission	admission	Sensitivity	Specificity
*Clinical parameters*					
Abnormal bleeding	Yes	5	1	10.2 (3.8–2.3)	98.2 (89.0–99.9)
	No	44	54		
Acidotic breathing	Yes	6	0	12.2 (5.1–25.5)	100 (91.9–100)
	No	43	55		
Cerebral malaria	Yes	13	1	26.5 (15.4–41.3)	98.2 (89.0–99.9)
	No	36	54		
Dark color urine	Yes	13	5	26.5 (15.4–41.3)	90.9 (79.3–96.6)
	No	36	50		
Pulmonary edema	Yes	18	3	36.7 (23.8–51.7)	94.5 (83.9–98.6)
	No	31	52		
Shock	Yes	19	8	38.8 (25.5–53.8)	85.5 (72.8–93.1)
	No	30	47		
Jaundice with organ dysfunction	Yes	37	30	75.5 (60.8–86.2)	45.5 (32.2–59.3)
	No	12	25		
*Laboratory parameters*					
Hypoglycemia	Yes	2	0	4.1 (0.7–15.1)	100 (91.9–100)
	No	47	55		
Severe anemia	Yes	2	1	4.1 (0.7–15.1)	98.2 (89.0–99.9)
	No	47	54		
Metabolic acidosis	Yes	16	1	32.7 (20.1–47.7)	98.2 (89.0–99.9)
	No	33	54		
Renal failure	Yes	17	3	34.7 (22.1–49.7)	94.5 (83.9–98.6)
	No	32	52		
Hemoglobinuria	Yes	24	27	49.0 (34.6–63.5)	50.9 (37.2–64.5)
	No	25	28		
Hyperparasitemia	Yes	24	20	49.0 (34.6–63.5)	63.6 (49.5–75.9)
	No	25	35		
≥3 WHO criteria	Yes	38	10	79.2 (64.6–89.0)	81.8 (68.7–90.5)
	No	11	45		

**Note:** WHO = World Health Organization; ICU = intensive care unit.

### Univariate and Multivariate Analysis for Requiring ICU in Patients with Severe Falciparum Malaria

In this study, no in-hospital mortality among patients with falciparum malaria was observed, thus, the surrogate endpoint of ICU admission was used for determining severity of malaria in patients. The median (IQR) for the duration of ICU admission in patients with severe falciparum malaria was 4.0 (2.0–4.0) days. Patients with severe falciparum malaria who had ethnicity of Thai or Myanmar, referred from other hospitals, no a prior history of malaria, and serum albumin <3.5 g/dL were more likely to require ICU admission. Patients with severe falciparum malaria who were admitted at the ICU received higher total dosages of artesunate and higher adjusted total dosages of artesunate per kilogram of body weight compared to those without ICU admission. Further, patients with severe falciparum malaria who were admitted at the ICU had significantly longer fever and parasite clearance times, periods of hospitalization, and a significantly higher proportion of hospital acquired infections (Table 3) than those without ICU admission.

**Table 3:** Baseline characteristics, clinical and laboratory parameters, management and outcomes of hospitalized patients with severe falciparum malaria (ICU or without ICU admission).

	Patients with severe falciparum malaria					
Characteristics	ICU admission		Without ICU admission		*P-*value	
	n	no. (%)	n	no. (%)		
*Baseline characteristics*						
Age, median (IQR), years	49	28.0 (20.5–42.0)	55	25.0 (19.0–34.0)	0.165	
Gender (male)	49	32 (65.3)	55	41 (74.5)	0.383	
Ethnic group	49		55		0.019	
Thai		17 (34.7)		12 (21.8)		
Myanmar		21 (42.9)		16 (29.1)		
Others		11 (22.4)		27 (47.2)		
Area visited	44		48		0.706	
Thai-Myanmar border		40 (90.9)		45 (93.8)		
Others		4 (9.2)		3 (6.3)		
Referrals	49	34 (69.4)	55	17 (30.9)	<0.001	
No history of malaria	49	46 (93.9)	54	35 (64.8)	0.001	
*Clinical and laboratory findings*						
Temperature >38.5°C	49	31 (63.3)	55	34 (61.8)	1.000	
WBC >10×10^9^/µL	49	18 (36.7)	55	11 (20.0)	0.093	
Presence of schizonts	49	25 (51.0)	55	22 (40.0)	0.352	
Albumin <3.5 g/dL	49	46 (93.9)	55	31 (56.4)	<0.001	
*Management and outcomes*						
Total artesunate, median (IQR), mg	49	920 (820–1080)	55	680 (600–840)	<0.001	
Total artesunate, median (IQR), mg/kg	31	16.4 (13.8–20.7)	48	13.0 (10.5–16.8)	0.001	
FCT, median (IQR), hours	49	121.0 (88.0–171.8)	54	73.0 (49.9–99.0)	<0.001	
PCT, median (IQR), hours	49	90.0 (68.3–107.8)	54	70.8 (56.3–87.1)	0.008	
Hospitalization, median (IQR), days	49	10.0 (7.5–15.0)	55	6.0 (5.0–8.0)	<0.001	
Hospital acquired infection	49	38 (77.6)	55	7 (12.7)	<0.001	

**Note:** ICU = intensive care unit; IQR = interquartile range; WBC = white blood cell counts; FCT = fever clearance time; PCT = parasite clearance time.

We used a univariate logistic regression analysis to ascertain which of the baseline characteristics, clinical symptoms, and laboratory findings associated with the requirement of ICU admission in patients with severe falciparum malaria. All factors that were significantly associated with severe malaria requiring ICU admission were included in the univariate logistic regression analysis. The results identified the following factors associated with severe malaria requiring ICU admission: (1) referrals, (2) ethnicity of Thai or Myanmar, (3) no a prior history of malaria, and (4) serum albumin <3.5 g/dL (Table 4).

**Table 4:** Univariate and multivariate logistic regression analysis of baseline characteristic, as well as clinical and laboratory parameters for requirement of ICU in hospitalized patients with severe falciparum malaria.

Characteristics	Univariate analysis			Multivariate logistic regression analysis		
	n	OR (95%CI)	*P-* value	n	OR (95%CI)	*P-* value
Referrals	104		<0.001	103		0.013
No		1.000 (reference)			1.000 (reference)	
Yes		5.067 (2.200–11.670)			3.571 (1.306–9.762)	
Ethnic	104		0.022	103		0.049
Thai		3.477 (1.256–9.630)	0.016		3.601 (1.011–12.822)	0.048
Myanmar		3.222 (1.238–8.383)	0.016		3.610 (1.138–11.445)	0.029
Others		1.000 (reference)			1.000 (reference)	
No history of malaria	103		0.001	103		0.018
No		8.324 (2.281–30.377)			5.887 (1.354–25.594)	
Yes		1.000 (reference)			1.000 (reference)	
Albumin <3.5 g/dL	104		<0.001	103		0.005
No		1.000 (reference)			1.000 (reference)	
Yes		11.871 (3.288–42.855)			7.200 (1.802–28.759)	

**Note:** ICU = intensive care unit; OR = odds ratio; CI = confidence interval.

All parameters with a *P*≤0.2 in the univariate logistic regression analysis were then further analyzed by the stepwise multiple logistic regression analysis using the backward selection method in order to determine the independent clinical factors that associate with the requirement for ICU admission in patients with severe falciparum malaria. We found the following clinical factors to be independently associated with the requirement for ICU admission in patients with severe falciparum malaria (1) referred patients, (2) ethnicity of Thai or Myanmar, (3) no a prior history of malaria, and (4) serum albumin <3.5 g/dL (Table 4).

During ICU admission, shock is known to be one of the common complications that develop in patients with severe falciparum malaria and is mainly found in non-survivors [11]. We compared the baseline characteristics, clinical and laboratory parameters, management and outcomes among patients who had shock to those without shock. The results showed that the baseline characteristics, clinical and laboratory parameters, management and outcomes among patients with and without shock were similar (Table 5).

**Table 5:** Baseline characteristics, clinical parameters, management and outcomes of patients with severe falciparum malaria requiring ICU (with shock or without shock).

	Severe falciparum malaria patients required ICU				
Characteristics	With shock		Without shock		*P-*value
	n	no. (%)	n	no. (%)	
*Baseline characteristics*					
Age, median (IQR), years	19	27.0 (21.0–37.0)	30	28.5 (19.8–49.0)	0.551
Gender (male)	19	9 (47.4)	30	23 (76.7)	0.073
Ethnic group	19		30		0.072
Thai		4 (21.1)		13 (43.3)	
Myanmar		12 (63.2)		9 (30.0)	
Others		3 (15.8)		8 (26.7)	
Area visited	18		26		0.868
Thai-Myanmar border		17 (94.4)		23 (88.5)	
Others		1 (5.6)		3 (11.4)	
Referrals	19	12 (63.2)	30	22 (73.3)	0.664
No history of malaria	19	17 (89.5)	30	29 (96.7)	0.551
*Clinical and laboratory findings*					
Temperature >38.5°C	19	11 (57.9)	30	20 (66.7)	0.752
WBC >10×10^9^/µL	19	8 (42.1)	30	10 (33.3)	0.752
Presence of schizonts	19	12 (63.2)	30	13 (43.3)	0.289
Albumin <3.5 g/dL	19	19 (100)	30	27 (90.0)	0.273
*Management and outcomes*					
Total artesunate, median (IQR), mg	19	920 (840–1080)	30	880 (815–1080)	0.475
Total artesunate, median (IQR), mg/kg	13	16.7 (15.1–25.9)	18	15.9 (13.2–19.2)	0.106
FCT, median (IQR), hours	19	126.0 (91.2–176.0)	30	116.5 (78.7–171.4)	0.442
PCT, median (IQR), hours	19	87.5 (68.5–100.5)	30	91.0 (65.1–113.1)	0.473
Hospitalization, median (IQR), days	19	10.0 (7.0–16.0)	30	10.0 (7.8–15.0)	0.942
Hospital acquired infections	19	16 (84.2)	30	22 (73.3)	0.492

**Note:** ICU = intensive care unit; IQR = interquartile range; WBC = white blood cell counts; FCT = fever clearance time; PCT = parasite clearance time.

## Discussion

Falciparum malaria is a major cause of severe malaria and death amongst infected individuals. Previous studies show that the mortality rate in patients with severe falciparum malaria varied between 10–30% [11], [12] and the incidence of severe malaria appears to be increasing [13]. Early identification and the timely institution of appropriate treatments may well improve the outcomes and reduce the mortality of these patients. For instance, at the Hospital for Tropical Diseases in Bangkok, the majority of patients who present with malaria are treated with anti-malarial drugs at the time of diagnosis prior to admission and they are then closely monitored in an effort to avert dire outcomes such as death and severe morbidity. This hospital has an in-hospital mortality rate of <1% for patients admitted with malaria, and during our study period (2006–2012), there were no reported deaths due to malaria infection [14]. In an effort to further improve the management of patients with malaria, we conducted a retrospective case-control study to compare clinical factors and outcomes between patients with severe malaria and those with non-severe malaria and to identify clinical factors associated with the requirement for ICU admission in patients with severe falciparum malaria among hospitalized adults in Thailand.

Thus far, a number of studies from various parts of the world have attempted to identify clinical predictors for severity of falciparum malaria, but they have reported very diverse findings [15]. These differences may well be attributed to variations in study design, such as the inclusion of patients with infections due to chloroquine-resistant falciparum malaria and those who self-administer anti-malarial drugs, or because of differences in the characteristics of study populations, including differences in patient age and ethnicity (Asian or Caucasian), the extent of parasitemia, hemoglobin and platelet levels as well as white blood cell counts [8]–[10]. In addition, current information on the clinical factors that predicted severity of severe falciparum malaria amongst adults in Southeast Asia is limited.

In this study, we identified the following clinical characteristics and laboratory findings that were more likely to develop severe falciparum malaria: (1) referrals, (2) no a prior history of malaria, (3) a body temperature of >38.5°C, (4) white blood cells counts >10×10^9^/µL, (5) the presence of schizonts in peripheral blood smears, and (6) albumin levels of <3.5 g/dL. In addition, patients with severe falciparum malaria had longer duration of hospitalization and more likely to developed hospital acquired infections than those with non-severe malaria. However, all patients with falciparum malaria included in our study were treated with an artesunate-based regimen. Patients were given a dose of 2.4 mg/kg of artesunate at the time of diagnosis and at 12 and 24 h post diagnosis. Treatment was continued at a dose of 2.4 mg/kg per day until parasitemia disappeared in peripheral blood smears [1], [16]. Most of the patients with falciparum malaria in our study acquired the infection after visiting the Thai-Myanmar border and the parasite clearance time in patients with severe falciparum malaria was longer than 3 days (77 h), indicating that partial drug resistance might be endemic to this region. Previous studies of the epidemiology of malaria in Thailand showed that the sensitivity of *P. falciparum* to treatment with artemisinin and artemisinin derivatives are declining in the areas around the Thai-Cambodia and the Thai-Myanmar border [1], [2], [17].

Using the 2010 WHO criteria for diagnosing severe malaria, 104 patients had severe falciparum malaria in this study. The presence of clinical jaundice with evidence of other vital organ dysfunction (64.4%) was the most common complications followed by hemoglobinuria (49.0%), shock (26.0%), pulmonary edema (20.2%), renal impairment (19.2%), severe metabolic acidosis (16.3%), cerebral malaria (13.5%), abnormal spontaneous bleeding (5.8%), severe anemia (2.9%), and hypoglycemia (1.9%). In addition, severe falciparum malaria patients who met ≥3 of the WHO criteria had sensitivity and specificity for requiring ICU admission of 79.2% and 81.8%, respectively. The other clinical factors that were associated with the requirement for ICU admission in hospitalized patients with severe falciparum malaria included (1) referrals, (2) ethnicity of Thai or Myanmar, (3) no a prior history of malaria, and (4) serum albumin <3.5 g/dL.

Previous study showed that referred patients were at risk to develop severe malaria due to delay in diagnosis and late effective treatment [18]. In addition, another study showed that no a prior history of malaria and Asian ethnicity were also risk factors for severe malaria [9], [10]. Our study population included patients from different ethnic groups. The majority of our patients were Thai (90, 35.3%) or Myanmar (81, 31.8%) and the remaining patients (84, 32.9%) represented a variety of hill tribes and smaller ethnic groups, including Karen (43, 51.2%) and Mon (37, 44.0%), African (2, 2.4%), Cambodian (1, 1.2%), and Laos (1, 1.2%). In this study, ethnicity of Thai or Myanmar was one of the clinical factors that were found to be associated with severe malaria. The other associated factor was low albumin levels which probably resulted from a shift in hepatic protein biosynthesis during inflammation. During inflammation, the synthesis of proteins involved in acute inflammatory responses, such as c-reactive protein, coagulation factors, fibrinogen, and complement components are favored over the synthesis of albumin [4], [19].

Previous studies showed that shock (27–49%) was one of the common complications of severe malaria patients who were admitted at the ICU [4], [5], [11]. Our findings revealed that clinical as well as laboratory findings, management, and outcomes of severe falciparum malaria patients requiring ICU admission who developed shock were similar to those without shock.

### Conclusions

Our findings show that patients with falciparum malaria who present with (1) referred, (2) no a prior history of malaria, (3) body temperatures of >38.5°C, (4) white blood cell counts >10×10^9^/µL, (5) presence of schizonts in peripheral blood smears, and (6) albumin concentrations of <3.5 g/dL were more likely to develop severe malaria. Using ≥3 of the 2010 WHO criteria, the sensitivity and specificity of the criteria for requiring ICU admission were 79.2% and 81.8%, respectively. Multivariate analysis identified the following clinical factors as independent associated factors for severe malaria requiring ICU admission; (1) ethnicity of Thai and Myanmar, (2) referrals, (2) no a prior history of malaria, and (3) albumin concentrations of <3.5 g/dL. However, the clinical parameters, management and outcomes of severe falciparum malaria patients requiring ICU admission who developed shock and those without shock were similar.

Our findings are important for the clinical management of patients with malaria as it contributes to the early identification of these patients. Early identification and the timely initiation of appropriate treatments may well improve the outcomes and reduce the mortality of patients with severe falciparum malaria.
